# Cooked Adzuki Bean Reduces High-Fat Diet-Induced Body Weight Gain, Ameliorates Inflammation, and Modulates Intestinal Homeostasis in Mice

**DOI:** 10.3389/fnut.2022.918696

**Published:** 2022-06-09

**Authors:** Qingyu Zhao, Zhenyu Liu, Yiqing Zhu, Han Wang, Zijian Dai, Xuehao Yang, Xin Ren, Yong Xue, Qun Shen

**Affiliations:** ^1^College of Food Science and Nutritional Engineering, China Agricultural University, Beijing, China; ^2^National Center of Technology Innovation (Deep Processing of Highland Barley) in Food Industry, Beijing, China; ^3^National Engineering Research Center for Fruit and Vegetable Processing, Beijing, China; ^4^Cofco Nutrition and Health Research Institute Co., LTD., Beijing, China; ^5^Beijing Advanced Innovation Center for Food Nutrition and Human Health, Beijing Engineering and Technology Research Center of Food Additives, Beijing Technology and Business University, Beijing, China

**Keywords:** cooked adzuki bean, obesity, inflammation, metabolic endotoxemia, gut microbiota

## Abstract

Adzuki bean is widely consumed in East Asia. Although the positive effects of its biologically active ingredients on obesity have been confirmed, the role of whole cooked adzuki bean in preventing obesity and the relationship between the effects and gut microbiota remain unclear. Mice were fed either a low-fat diet (LFD) or high-fat diet (HFD) with or without 15% cooked adzuki bean for 12 weeks. Cooked adzuki bean significantly inhibited weight gain and hepatic steatosis, reduced high levels of serum triacylglycerol (TG), alanine aminotransferase (ALT), and aspartate aminotransferase (AST), and alleviated systemic inflammation and metabolic endotoxemia in mice fed a HFD. Importantly, cooked adzuki bean regulated gut microbiota composition, decreased the abundance of lipopolysaccharide (LPS)-producing bacteria (*Desulfovibrionaceae,Helicobacter*,and *Bilophila*), and HFD-dependent taxa (*Deferribacteraceae, Ruminiclostridium_9, Ruminiclostridium, Mucispirillum, Oscillibacter, Enterorhabdus, Tyzzerella, Anaerotruncus, Intestinimonas, unclassified_f_Ruminococcaceae, Ruminiclostridium_5*, and *Ruminococcaceae*), and enriched *Muribaculaceae, norank_f_Muribaculaceae, Anaeroplasma, Lachnospiraceae_NK4A136_group*, and *Lachnospiraceae* to alleviate inflammation and metabolic disorders induced by HFD. These findings provide new evidence for understanding the anti-obesity effect of cooked adzuki bean.

## Introduction

Obesity is becoming a worldwide health problem (1). The main feature of obesity is excessive fat accumulation, accompanied by dyslipidemia, liver injury, and hepatic steatosis. Excessive fat accumulation leads to adipocyte hypertrophy and the production of pro-inflammatory cytokines (tumor necrosis factor-α and interleukin-6). Therefore, obesity is also considered a type of chronic low-grade inflammation (2). The current treatment of obesity is often inefficient and accompanied by side effects, so effective and safe anti-obesity methods are needed. Dietary recommendations, such as legumes consumption, have received increasing attention because they prevent obesity by alleviating dyslipidemia, suppressing inflammatory signals, and reshaping gut microbiota (3).

Gut microbiota regulates many physiological processes through interaction with the host, and the abnormal function or composition of gut microbiota contribute to the development of metabolic diseases (4). Studies have shown that HFDs not only significantly increase body fat mass, but also reshape the gut microbiota (5). Gut microbiota dysbiosis induced by HFD increase energy collection and storage, destroy the intestinal barrier, cause inflammation, and lead to obesity (6). In addition, LPS is an important metabolite of gut microbiota, and its release is related to systemic inflammation and obesity (7). HFD feeding impairs intestinal integrity, promotes the transfer of LPS from intestinal microbiota to blood, and causes metabolic endotoxemia (8). Emerging evidence shows that diets can quickly and reproducibly change the structure of gut microbiota (9). Therefore, dietary intervention is considered to be an effective way to alleviate gut microbiota dysbiosis.

Adzuki bean is commonly consumed in East Asia and is treated as a diuretic and an antidote (10). Adzuki bean is rich in biologically active ingredients, such as flavonoids, proanthocyanidins, saponins, and functional polypeptide (11, 12). Previous studies have shown that saponins and flavonoids from adzuki bean prevent HFD-induced obesity in mice (13). Adzuki bean resistant starch reduced serum cholesterol level in rats fed a cholesterol diet (14). Adzuki bean dietary fiber reduced visceral fat accumulation in rats fed a normal diet (15). The biologically active peptides in adzuki bean exhibited significant anti-inflammatory activity by inhibiting the expression of tumor necrosis factor-α (TNF-α) and interleukin-6 (IL-6) (16). In addition, adzuki bean water extract containing polyphenols attenuated obesity by regulating gut microbiota (17). Most adzuki bean studies on reducing obesity induced by HFD focused mainly either on the effects of bean extracts or on single chemicals. However, people in daily life usually consume adzuki bean as whole beans and make it into porridge, bean paste, and soup. Beans are usually not consumed raw and need to be processed before consumption. Processing can promote protein digestion by reducing the level of antinutritional factors in beans (18). Importantly, Processing not only significantly decreased the content of total polyphenols and total flavonoids in adzuki bean, but also significantly reduced the bioavailability of total phenols and antioxidant activity (19–21). Compared with the peptides obtained from raw adzuki bean protein, the thermally processed peptides showed significantly reduced inhibition of 3-hydroxy-3-methylglutaryl coenzyme-A reductase catalytic activity (22). Our previous study has found that 15% raw adzuki bean supplementation significantly alleviated obesity in HFD-fed mice (23). We also found that heat-treated adzuki bean protein hydrolysates reduced obesity in mice fed a HFD *via* remodeling gut microbiota (12). But it is of more practical importance for dietary guidance to understand if cooked adzuki bean can prevent obesity. Therefore, it is also necessary to clarify the relationship between the effects of cooked adzuki bean on preventing obesity and gut microbiota.

Thus, we assessed the effects of cooked adzuki bean on the body weight gain, serum lipids, and systemic inflammation of mice fed a HFD, and further analyzed the changes in gut microbiota composition. The present study reveals the link between the gut microbiota and cooked adzuki bean-mediated metabolic improvement. Results may help to understand the mechanism of cooked adzuki bean in preventing obesity, especially the role of the gut microbiota.

## Materials and Methods

### Material Preparation

Adzuki bean from Dongfangliang Life Technology Co., Ltd., (Datong, China) was soaked for 12 h, steamed for 2 h, and dried for 12 h at 40°C. Cooked beans were pulverized into 80-mesh powders. The basic nutritional composition of cooked adzuki bean was showed in Supplementary Table 1.

### Animal Experiment

**FIGURE 1:**
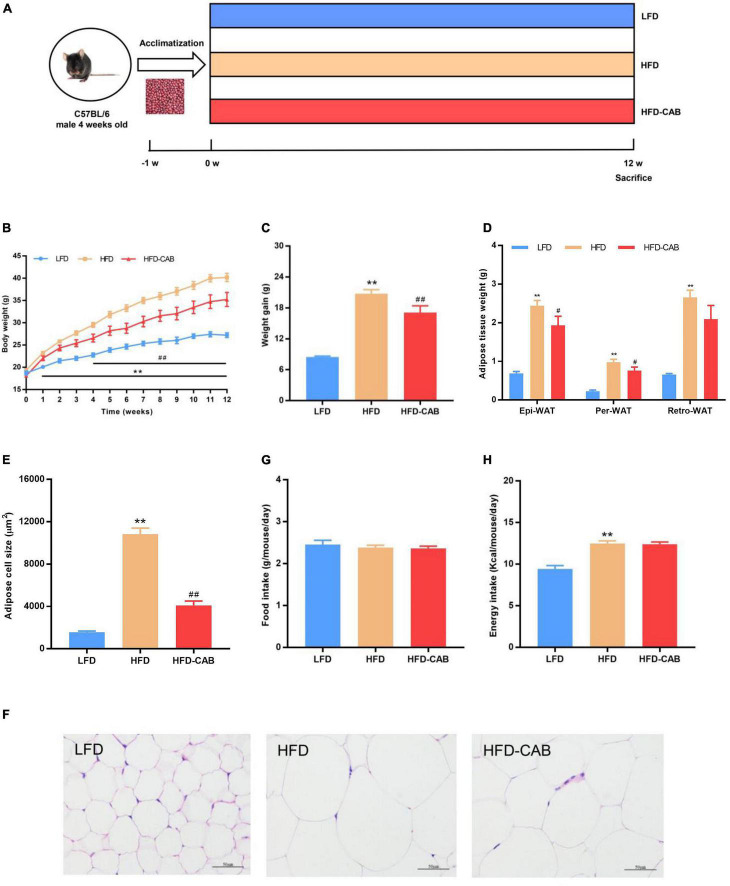
Cooked adzuki bean reduces HFD-induced body weight gain and lipid accumulation in mice. **(A)** Experimental protocol and design, **(B)** Body weight time courses, **(C)** Weight gain, **(D)** Adipose tissue weight, **(E)** Adipose cell size, **(F)** H&E staining of Epi-WAT sections, **(G)** Food intake, and **(H)** Energy intake. Data presented as mean ± SEM, *n* = 7 per group. **P < 0.01 vs. LFD; ^#^*P* < 0.05 and ^##^*P* < 0.01 vs. HFD. Epi-WAT epididymal fat; Per-WAT perirenal fat; Retro-WAT retroperitoneal fat; LFD, low-fat diet; HFD, high-fat diet; HFD-CAB, high-fat diet supplemented with cooked adzuki bean.

All experimental design was approved by the Animal Care Committee of China Agricultural University (AW09089102-4). Four-week-old male C57BL/6 mice (Vital River Laboratory Animal Technology Co., Ltd., Beijing, China) were housed in a Specific-Pathogen-Free facility with controlled conditions (55 ± 5% humidity, 24 ± 2°C, 12 h light-dark cycle) with free access to water and food. After 1 week of adaptive feeding, mice were randomly divided into three groups (*n* = 7 per group) and housed at 2 or 3 per cage. The design of the 12-week animal experiment (Figure 1A) was as follows: (1) low-fat diet (LFD, 3.85 total kcal/g, 10% kcal from fat), (2) high-fat diet (HFD, 5.24 total kcal/g, 60% kcal from fat), and (3) HFD supplemented with 15% cooked adzuki bean powder (HFD-CAB, 5.24 total kcal/g, 60% kcal from fat). The detailed diet energy densities and compositions were determined (Supplementary Table 2). The LFD (D12450J) and HFD (D12492) diets were obtained from Research Diets Inc. (New Brunswick, NJ, United States). Daily intake of 200–300 g of cereal-grain, including 50–150 g of whole grain and pulses, is recommended according to the Dietary Guidelines for Chinese Residents (24). The dietary guidelines suggest a 50% maximum intake level for pulses. However, it is difficult for people to consume so many legumes daily, so a more practical low intake level needs to be recommended. The 15% cooked adzuki bean supplementation level refers to previous studies (15, 23, 25) and is an physiologically relevant and achievable level of pulse intake in humans (∼1 cup/day) (26). When whole grain and pulses are added to animal feed, the diet must be adjusted to maintain the original composition of whole grain and pulses and ensure the consistency of energy intake (27). The composition of HFD-CAB diet was matched to the HFD diet based on previous studies (26). Importantly, the HFD-CAB and HFD diets did not differ in macronutrient composition and were both isocaloric, so each macronutrient contributed the same to the caloric density of the diet. Body weight and food intake was measured once a week. After 12-weeks, fecal samples were collected and the mice were sacrificed after a 12-h overnight fast. Epididymal, perirenal, and retroperitoneal fat were rapidly removed and weighed.

### Biochemical Analysis

Blood samples were collected from the orbital vascular plexus and centrifuged at 3,000 rpm at 4°C for 10 min to obtain serum. The TG, total cholesterol (TC), high-density lipoprotein cholesterol (HDL-C), low-density lipoprotein cholesterol (LDL-C), ALT, and AST were measured with an automatic biochemistry analyzer (Hitachi Ltd., Tokyo, Japan).

### Histological Analysis

Samples of adipose and liver tissue were fixed in 4% paraformaldehyde, and then stained with hematoxylin and eosin (H&E). The liver samples were also stained with Oil Red O solution.

### Inflammatory Factors and LPS Determination

The serum concentration of TNF-α and IL-6 were measured with enzyme-linked immunosorbent assay kits from Nanjing Jiancheng Bioengineering Institute. According to the manufacturer’s protocol, LPS was measured with enzyme-linked immunosorbent assay kit from Shanghai Enzyme-linked Biotechnology Co., Ltd.

### 16S rRNA Gene Sequencing Analysis

According to the manufacturer’s protocol, microbial community genomic DNA was extracted from fecal samples using the E.Z.N.A. soil DNA Kit. The DNA purity and concentration were determined with a NanoDrop 2000 UV-vis spectrophotometer. The hypervariable region V3–V4 of the bacterial 16S rRNA gene was amplified with primer pairs 338F (5′-ACTCCTACGGGAGGCAGCAG-3′) and 806R (5′-GGACTACHVGGGTWTCTAAT-3′) with an ABI GeneAmp 9700 PCR thermocycler. The PCR product was extracted from 2% agarose gel, then purified and quantified. High-throughput sequencing was done by Majorbio (Shanghai Majorbio Bio-pharm Technology Co., Ltd., China).

### Statistical Analysis

The data were shown as mean ± SEM. Group differences were determined by One-Way ANOVA followed by Duncan test using SPSS 22.0 (SPSS Inc., Chicago, IL, United States) where *P* < 0.05 was considered statistically significant. Prism 5 (La Jolla, CA, United States) was used to prepare graphs.

## Results

### Cooked Adzuki Bean Reduces Body Weight Gain and Fat Accumulation in Mice Fed a High-Fat Diet

At week 0, no significant difference occurred in body weight among the three groups (Figure 1B). After 12-weeks, mice in HFD group showed higher body weight compared with the LFD group, and mice in the HFD-CAB group had significantly lower body weight than the HFD group (Figure 1B, *P* < 0.01). Consistently, the weight gain of HFD-CAB group mice was significantly lower than that in the HFD group (Figure 1C, *P* < 0.01). Weight gain is usually accompanied by fat accumulation. HFD feeding led to a significant increase in white adipose tissue (Per-WAT, Epi-WAT, and Retro-WAT) weight, while cooked adzuki bean significantly reduced the weight of Epi-WAT and Per-WAT compared to the HFD group (Figure 1D, *P* < 0.05). In addition, HFD feeding significantly promoted adipocyte hypertrophy and increased adipocyte area, while cooked adzuki bean effectively inhibited the increase of adipocyte size compared with the HFD group (Figures 1E,F, *P* < 0.01). These results indicate that cooked adzuki bean reduces fat accumulation and body weight gain in HFD-fed mice. The inhibitory effects of cooked adzuki bean are not due to reduced food consumption, because no significant differences occurred in energy intake and food intake between the HFD-CAB and HFD groups (Figures 1G,H, *P* > 0.05).

### Cooked Adzuki Bean Alleviates Dyslipidemia and Liver Injury in High-Fat Diet-Fed Mice

**FIGURE 2:**
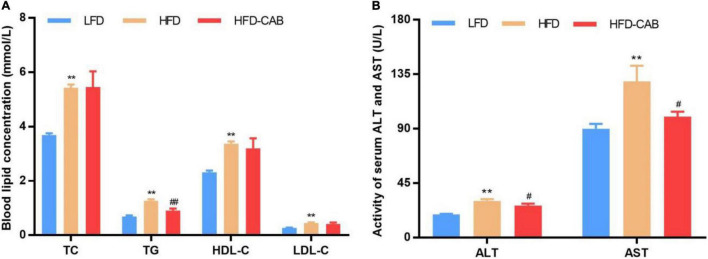
Cooked adzuki bean alleviates dyslipidemia and liver injury in HFD-fed mice. **(A)** Serum lipid profile, and **(B)** Serum ALT and AST levels. Data presented as mean ± SEM, *n* = 7 per group. **P < 0.01 vs. LFD; ^#^*P* < 0.05 and ^##^*P* < 0.01 vs. HFD. TC total cholesterol; TG triacylglycerol; HDL-C high-density lipoprotein cholesterol; LDL-C low-density lipoprotein cholesterol; AST aspartate aminotransferase; ALT alanine aminotransferase; LFD, low-fat diet; HFD, high-fat diet; HFD-CAB, high-fat diet supplemented with cooked adzuki bean.

As expected, HFD feeding induced dyslipidemia, manifested by significantly increased serum TC, TG, HDL-C, and LDL-C levels, while cooked adzuki bean led to a significant decrease in serum TG level (Figure 2A, *P* < 0.01). The liver injury of HFD group mice was indicated by significantly elevated serum AST and ALT levels. In contrast, compared with the HFD group cooked adzuki bean significantly reduced serum AST and ALT levels (Figure 2B, *P* < 0.05). These results indicate that cooked adzuki bean significantly ameliorates the high level of serum TG and liver injury in HFD-fed mice.

### Cooked Adzuki Bean Limits High-Fat Diet-Induced Hepatic Steatosis

**FIGURE 3:**
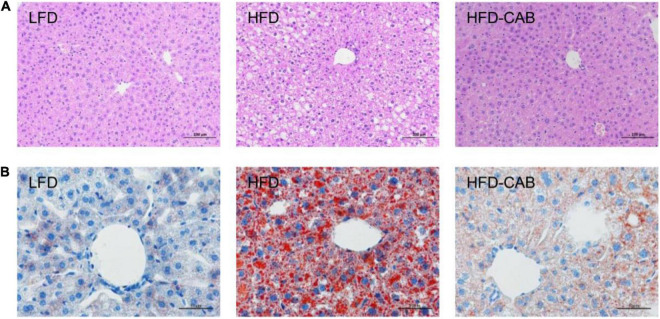
Cooked adzuki bean limits HFD-induced hepatic steatosis. **(A)** H&E staining of liver tissue sections, and **(B)** Liver Oil Red O staining. *n* = 7 per group. LFD, low-fat diet; HFD, high-fat diet; HFD-CAB, high-fat diet supplemented with cooked adzuki bean.

Histological analysis showed that HFD feeding caused lipid accumulation in mouse liver compared with the LFD group, accompanied by a large number of cytoplasmic vacuoles and hepatocyte swelling, whereas cooked adzuki bean reduced lipid accumulation and effectively inhibited hepatic steatosis (Figure 3).

### Cooked Adzuki Bean Ameliorates Inflammation and Metabolic Endotoxemia in High-Fat Diet-Fed Mice

**FIGURE 4:**
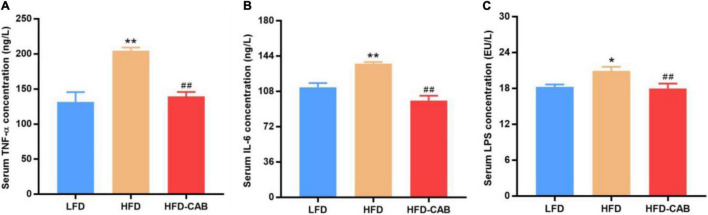
Cooked adzuki bean ameliorates inflammation and metabolic endotoxemia in HFD-fed mice. **(A)** Serum tumor necrosis factor-α (TNF-α) concentrations, **(B)** Serum interleukin-6 (IL-6) concentrations, and **(C)** Serum lipopolysaccharide (LPS) levels. Data presented as mean ± SEM, *n* = 7 per group. *P < 0.05 and **P < 0.01 vs. LFD; ^##^*P* < 0.01 vs. HFD. LFD, low-fat diet; HFD, high-fat diet; HFD-CAB, high-fat diet supplemented with cooked adzuki bean.

As expected, compared with the LFD group HFD feeding caused significant increase in serum IL-6, TNF-α, and LPS in mice. In contrast, cooked adzuki bean maintained normal levels of IL-6, TNF-α, and LPS (Figure 4, *P* < 0.01). These results indicate that cooked adzuki bean significantly reduced HFD-induced inflammation and metabolic endotoxemia in mice.

### Cooked Adzuki Bean Modulates α and β Diversity of Gut Microbiota in Mice Fed a High-Fat Diet

**FIGURE 5:**
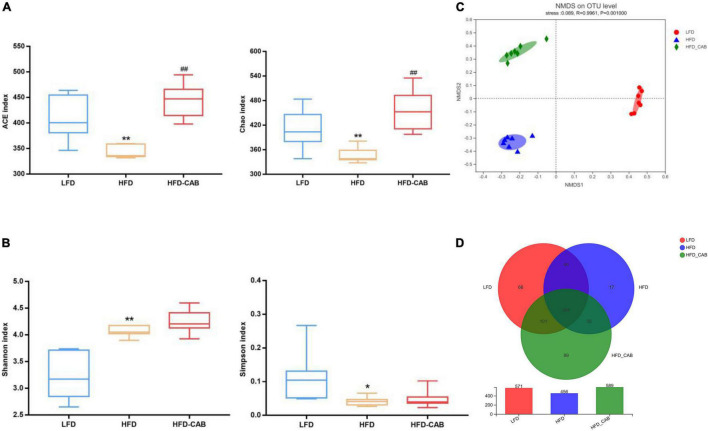
Cooked adzuki bean modulates α and β diversity of gut microbiota in HFD-fed mice. **(A)** The community richness accessed by the ACE and Chao indexes, **(B)** The community diversity accessed by the Shannon and Simpson indexes, **(C)** Non-metric multidimensional scaling (NMDS) score plot based on Bray–Curtis, and **(D)** Venn diagram showing the OTUs shared by the mice from each group. Data presented as mean ± SEM, *n* = 7 per group. *P < 0.05 and **P < 0.01 vs. LFD; ^##^*P* < 0.01 vs. HFD. LFD, low-fat diet; HFD, high-fat diet; HFD-CAB, high-fat diet supplemented with cooked adzuki bean.

More and more studies show that the gut microbiota is involved in the development of obesity and related diseases (28). Therefore, 16S rRNA high-throughput sequencing was applied to explore the effects of cooked adzuki bean on gut microbiota of mice fed a HFD. Alpha diversity is represented by the community richness (Chao and ACE indexes) and community diversity (Simpson and Shannon indexes). HFD feeding resulted in significantly decreased ACE, Chao, and Simpson indexes, and an increased Shannon index compared with the LFD group (Figures 5A,B, *P* < 0.05). In contrast, cooked adzuki bean significantly modulated the decline of ACE and Chao indexes induced by HFD, indicating that cooked adzuki bean significantly improved the community richness. To learn more about the influence of cooked adzuki bean on the structure and composition of gut microbiota, β diversity based on the NMDS score plot was performed. The more similar the gut microbiota composition of samples, the closer the distance in the NMDS diagram. The results showed that cooked adzuki bean had a significant effect on the gut microbiota composition and modulated the overall structure of gut microbiota damaged by HFD (Figure 5C). Venn diagram analysis was performed to understand the differences of gut microbiota in all groups of mice (Figure 5D). In the three groups of mice, 364 identical OTUs were identified. The LFD, HFD, and HFD-CAB groups showed 66, 17, and 89 unique OTUs, respectively.

### Cooked Adzuki Bean Modulates Microbial Taxonomic Profiles in High-Fat Diet-Fed Mice

**FIGURE 6:**
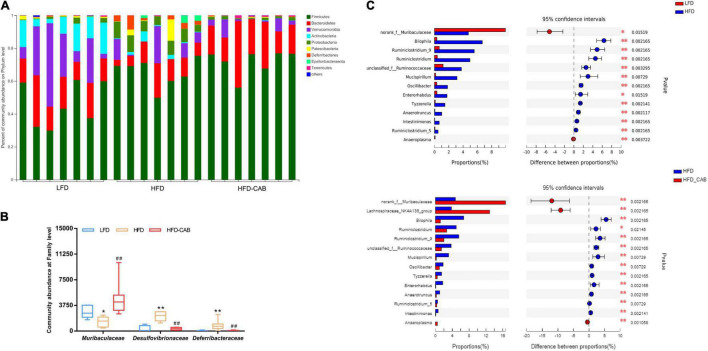
Cooked adzuki bean modulates microbial taxonomic profiles in HFD-fed mice. **(A)** Bacterial profile at the phylum level, **(B)** Relative abundance of *Muribaculaceae, Desulfovibrionaceae*, and *Deferribacteraceae* at the family level, and **(C)** Mean proportions of key genera in different groups. Data presented as mean ± SEM, *n* = 7 per group. *P < 0.05 and **P < 0.01 vs. LFD; ^##^*P* < 0.01 vs. HFD **(B).** *P < 0.05 and **P < 0.01 **(C)**. LFD, low-fat diet; HFD, high-fat diet; HFD-CAB, high-fat diet supplemented with cooked adzuki bean.

At the phylum level, the gut microbiota mainly includes *Firmicutes, Bacteroidetes, Verrucomicrobia, Actinobacteria*, and *Proteobacteria* (Figure 6A). At the family level, HFD feeding significantly enriched *Desulfovibrionaceae* and *Deferribacteraceae*, and decreased the abundance of *Muribaculaceae* (Figure 6B, *P* < 0.05). However, cooked adzuki bean significantly alleviated the changes in gut microbiota induced by HFD. At the genus level, the HFD group showed significant enrichment of *Bilophila, Ruminiclostridium_9, Ruminiclostridium, unclassified_f_Ruminococcaceae, Mucispirillum, Oscillibacter, Enterorhabdus, Tyzzerella, Anaerotruncus, Intestinimonas, Ruminiclostridium_5*, and a reduction in *norank_f_Muribaculaceae* and *Anaeroplasma* (Figure 6C). Cooked adzuki bean not only alleviated these changes, but also significantly increased the abundance of *Lachnospiraceae_NK4A136_group*. In short, cooked adzuki bean relieved the disorders of gut microbiota composition induced by HFD, contributing to the anti-obesity effect.

### Effect of Cooked Adzuki Bean on Key Phylotypes

**FIGURE 7:**
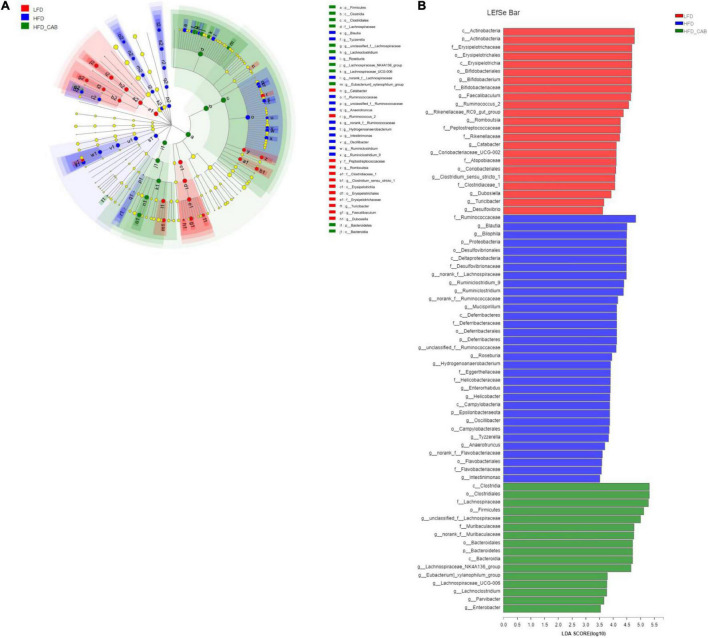
Effect of cooked adzuki bean supplementation on key phylotypes. **(A)** Cladogram generated from LEfSe analysis showing the relationship between taxon (the levels represent, from the inner to outer rings, phylum, class, order, family, and genus), and **(B)** linear discriminant analysis (LDA) scores derived from LEfSe analysis (the length of the bar represents the LDA score).

LEfSe analysis was used to further screen bacteria enriched in different dietary groups to obtain a clade map and an LDA value distribution histogram. The levels of specific bacterial taxa varied from phylum to genus in each group (Figure 7A). LEfSe analysis indicated that *Ruminococcaceae* and *Helicobacter* had higher abundance in HFD group mice. The HFD-CAB group was characterized by a higher abundance of *Lachnospiraceae, norank_f_Muribaculaceae, Muribaculaceae*, and *Lachnospiraceae_NK4A136_group* (Figure 7B).

### Correlation Between Gut Microbiota and Obesity-Related Indicators

**FIGURE 8:**
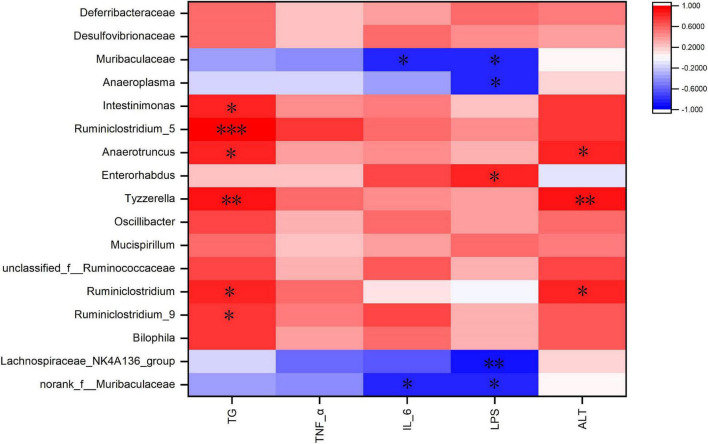
Heatmap of Spearman’s correlation between the gut microbiota and obesity-related indices. The intensity of the colors represented the degree of association (red, positive correlation; blue, negative correlation). Significant correlations are marked by *P < 0.05; **P < 0.01; ***P < 0.001.

Spearman correlation analysis was used to identify the main gut microbiota affecting obesity-related indicators (Figure 8). The heat map shows that TG was positively correlated with *Ruminiclostridium_5, Ruminiclostridium_9, Ruminiclostridium, Tyzzerella, Anaerotruncus*, and *Intestinimonas*. ALT was positively correlated with *Ruminiclostridium, Tyzzerella*, and *Anaerotruncus*. IL-6 was negatively correlated with *Muribaculaceae* and *norank_f_Muribaculaceae* (Figure 8). LPS was negatively correlated with *Muribaculaceae, norank_f_Muribaculaceae, Anaeroplasma*, and *Lachnospiraceae_NK4A136_group*, and was positively correlated with *Enterorhabdus*. These bacteria may be critical to the development of obesity and its complications.

## Discussion

At present, the incidence of obesity is increasing worldwide (1). HFDs promote the development of obesity, yet there is growing evidence that legume consumption can alleviate obesity (29). In this study, cooked adzuki bean significantly inhibited body weight gain, reduced serum TG and proinflammatory cytokines (IL-6, TNF-α, and LPS) levels, and alleviated obesity-related hepatic steatosis and liver injury. Importantly, the beneficial effects of cooked adzuki bean may be closely related to the improvement of gut microbiota imbalances. Evidence shows that legume consumption alleviates obesity by reducing weight gain and serum lipid levels (23). Consistent with previous studies, cooked adzuki bean significantly inhibited weight gain and reduced serum TG (Figures 1B, 2A). Interestingly, cooked adzuki bean had no effect on TC, HDL-C, and LDL-C (Figure 2A). A previous study found that heat treatment resulted in a significant reduction in the cholesterol-lowering properties of adzuki bean peptides (22). Long-term lipid metabolism disorders lead to liver injury. AST and ALT are likely the most sensitive indicators of liver function. It was shown previously that adzuki bean hot water extract containing polyphenols significantly alleviated HFD-induced liver injury (17). Similarly, cooked adzuki bean significantly reduced the levels of serum AST and ALT (Figure 2B). Due to extensive lipid accumulation in hepatocytes, obesity is often accompanied by hepatic steatosis, which also promotes the development of nonalcoholic fatty liver disease (NAFLD) (30). Previous studies found that adzuki bean supplementation attenuated lipid accumulation by inhibiting the expression of mRNA related to adipogenesis in the liver of NAFLD model mice (25). As expected, cooked adzuki bean also alleviated hepatic steatosis induced by HFD (Figure 3). These important beneficial effects of cooked adzuki bean on obese mice were not due to reduced food consumption, since no significant differences occurred in energy intake and food intake between the HFD and HFD-CAB groups (Figures 1G,H).

HFDs lead to hyperlipidemia, which in turn promotes the occurrence of inflammation. Inflammation is one of the main mechanisms affecting the development of obesity. TNF-α can be produced by adipocytes, and promote the production of IL-6 by activating the MAPK and NF-κB signaling pathways (31). IL-6 induces inflammation by affecting the secretory function of intestinal epithelial cells (32). It was shown previously that adzuki bean supplementation relieved inflammation by inhibiting the expression of proinflammatory mediators (TNF-α, caspase-3, and nuclear factor κB) in the liver of NAFLD model mice (25). In addition, adzuki bean peptides exhibited significant anti-inflammatory activity by inhibiting the expression of TNF-α and IL-6 (16). Adzuki bean extract containing polyphenols also led to a reduction in proinflammatory adipocytokines in human adipocytes (33). In this study, cooked adzuki bean significantly reduced serum IL-6 and TNF-α (Figures 4A,B). LPS is connected with elevated levels of TNF-α and IL-6, and can increase intestinal permeability and induce inflammation through the Toll-like receptor 4 signaling pathway (34, 35). HFDs lead to the increased serum LPS in human (36). Cooked adzuki bean significantly inhibited the high level of LPS induced by HFD, which may contribute to the alleviation of inflammation (Figure 4C). Previous studies have found that adzuki bean ethanol extract decreased the concentrations of LPS and various circulating proinflammatory cytokines such as IL-1β, TNF-α, and IL-6 in HFD-fed mice (37).

Many studies have shown that HFDs promote the development of obesity by inducing gut microbiota dysbiosis (38). As a shared substrate between the host and gut microbiota, diets have been reported to manage obesity by regulating the composition of the gut microbiota (39). Microbial diversity, including α diversity and β diversity, is an important biomarker in the obesity development (40). Cooked adzuki bean significantly inhibited the HFD-mediated decrease in ACE and Chao indexes (Figure 5A). Obese individuals with lower community richness have more weight gain over time (41). It has been reported that adzuki bean dietary fiber significantly increased the number of observed species (15). β diversity analysis also confirmed that cooked adzuki bean changed the overall structure of gut microbiota (Figure 5C). Previous studies have shown that both adzuki bean polyphenolic extract and heat-treated protein hydrolysates have significant effects on gut microbial community composition (12, 17).

*Ruminococcaceae* promotes fat synthesis (42). *Ruminiclostridium_9* leads to abnormal lipid metabolism (43). In contrast, *Lachnospiraceae_NK4A136_group* is involved in the regulation of body weight (44). *Norank_f_Muribaculaceae* relieves abnormal lipid metabolism (23). In the present study, TG was positively correlated with *Ruminiclostridium_5, Ruminiclostridium_9, Ruminiclostridium, Tyzzerella, Anaerotruncus*, and *Intestinimonas* (Figure 8). Cooked adzuki bean significantly reduced the abundance of *Ruminococcaceae, Ruminiclostridium_5, Ruminiclostridium_9, Ruminiclostridium, Tyzzerella, Anaerotruncus*, and *Intestinimonas*, and increased the abundance of *Lachnospiraceae_NK4A136_group* and *norank_f_Muribaculaceae*, which may contribute to the alleviation of body weight gain and reduction in the level of serum TG (Figures 1B–F, 2A). It has been found that soybean insoluble dietary fiber significantly increased the abundance of *Lachnospiraceae_NK4A136_group* (45). Previous studies have also shown that raw adzuki bean significantly enriched the occurrence of *norank_f_Muribaculaceae* and *Lachnospiraceae_NK4A136_group*, and returned HFD-dependent taxa (*Ruminiclostridium_9* and *Ruminiclostridium*) back to normal status (23). Interestingly, raw adzuki bean supplementation enriched the abundance of *Bifidobacterium* in obese mice, but this effect did not appear after cooked adzuki bean supplementation (23). Studies have found that polyphenols significantly increase the abundance of *Bifidobacterium*, because polyphenols are beneficial to the growth, proliferation or survival of gut microbiota (46, 47). A previous study found that processing caused a significant decrease in the adzuki bean polyphenols, which may be due to high temperature and the destruction of bean tissues during cooking (19, 48). Therefore, the difference of *Bifidobacterium* between raw adzuki bean and cooked adzuki bean supplementation may be due to the loss of adzuki bean polyphenols after processing.

HFDs can increase intestinal permeability and promote the entrance of inflammatory mediators into systemic circulation, thereby inducing systemic inflammation (49). *Deferribacteraceae, Enterorhabdus, Anaerotruncus*, *Intestinimonas*, *Ruminiclostridium_5*, and *Ruminiclostridium_9* promote the development of inflammation (43, 50–54). In addition, *Tyzzerella* and *unclassified_f_Ruminococcaceae* are positively correlated with pro-inflammatory cytokines (55, 56). In contrast, *Lachnospiraceae_NK4A136_group, Lachnospiraceae*, *norank_f_Muribaculaceae*, and *Anaeroplasma* have anti-inflammatory effects (57–60). In this study, IL-6 was negatively correlated with *Muribaculaceae* and *norank_f_Muribaculaceae* (Figure 8). Cooked adzuki bean significantly decreased the abundance of *Deferribacteraceae, Enterorhabdus, Anaerotruncus*, *Intestinimonas*, *Ruminiclostridium_5, Ruminiclostridium_9, Tyzzerella*, and *unclassified_f_Ruminococcaceae*, and enriched the *Lachnospiraceae_NK4A136_group, Lachnospiraceae*, *norank_f_Muribaculaceae*, *Anaeroplasma*, and *Muribaculaceae*, all of which may contribute to the alleviation of inflammation (Figures 4A,B). It has been reported that heat-treated adzuki bean protein hydrolysates significantly increased the abundance of *Anaeroplasma* in mice fed a HFD (12). Mung bean protein supplementation can significantly enrich *Muribaculaceae* (61).

Gut microbiota is the part of intestinal barrier and plays a key role in maintaining intestinal health. When leaky gut occurs, harmful bacteria and their metabolites (e.g., LPS) invade the intestinal and circulatory system, causing the release of proinflammatory cytokines and leading to metabolic endotoxemia (62). HFD feeding increases the circulating LPS concentration, which is closely related to obesity-related inflammation (63). *Desulfovibrionaceae, Helicobacter*, and *Bilophila* produce LPS (64–67). In addition, *Mucispirillum* and *Ruminococcaceae* damage the intestinal mucosa (68, 69). *Oscillibacter* and *Ruminiclostridium* are related to intestinal dysfunction (70–73). In contrast, *Muribaculaceae* is beneficial to the health of the intestinal epithelium (74). In this study, LPS was negatively correlated with *Muribaculaceae, norank_f_Muribaculaceae, Anaeroplasma*, and *Lachnospiraceae_NK4A136_group*, and was positively correlated with *Enterorhabdus* (Figure 8). Cooked adzuki bean significantly reduced the abundance of LPS-producing bacteria (*Desulfovibrionaceae*,*Helicobacter*,*Bilophila*), *Mucispirillum*, *Ruminococcaceae, Oscillibacter, Ruminiclostridium*, and *Enterorhabdus*, and enriched *Muribaculaceae, norank_f_ Muribaculaceae, Anaeroplasma*, and *Lachnospiraceae_NK4A136_ group*, which have contributed to the reduction of serum LPS and the maintenance of intestinal health (Figure 4C). Similarly, heat-treated adzuki bean protein hydrolysates significantly decreased the abundance of *Bilophila* and *Mucispirillum* in HFD-fed mice (12).

**FIGURE 9:**
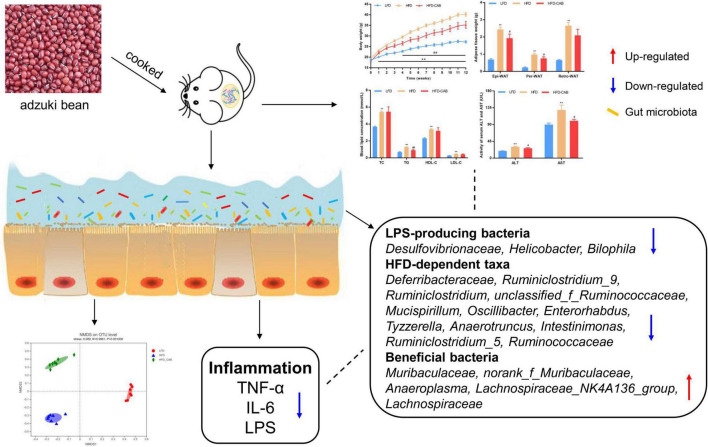
Schematic representation of the anti-obesity effect of cooked adzuki bean *via* gut microbiota.

In conclusion, cooked adzuki bean showed beneficial effects on HFD-induced obesity in mice by inhibiting body weight gain, reducing the levels of serum TG, ALT, and AST, and limiting hepatic steatosis (Figure 9). Of particular significance, cooked adzuki bean also improved systemic inflammation and metabolic endotoxemia, and maintained intestinal health, indicating that adzuki bean is an effective functional food for preventing obesity and related complications. Yet to be clarified is the relationship between the positive effects of cooked adzuki bean on obesity and the adzuki bean-mediated changes of gut microbiota composition. Consequently, further work is needed to elucidate the specific mechanisms of the beneficial effects of cooked adzuki bean supplementation.

## Data Availability Statement

The original contributions presented in this study are included in the article/Supplementary Material, further inquiries can be directed to the corresponding author.

## Ethics Statement

The animal study was reviewed and approved by the Animal Care Committee of China Agricultural University (AW09089102-4).

## Author Contributions

QZ: conceptualization, methodology, investigation, data curation, and writing – original draft. ZL, YZ, HW, ZD, XY, XR, and YX: supervision. QS: writing – review and editing and funding acquisition. All authors contributed to the article and approved the submitted version.

## Conflict of Interest

XY was employed by the Cofco Nutrition and Health Research Institute Co., Ltd. The remaining authors declare that the research was conducted in the absence of any commercial or financial relationships that could be construed as a potential conflict of interest.

## Publisher’s Note

All claims expressed in this article are solely those of the authors and do not necessarily represent those of their affiliated organizations, or those of the publisher, the editors and the reviewers. Any product that may be evaluated in this article, or claim that may be made by its manufacturer, is not guaranteed or endorsed by the publisher.
