# Supplementary material for: Cooked Adzuki Bean Reduces High-Fat Diet-Induced Body Weight Gain, Ameliorates Inflammation, and Modulates Intestinal Homeostasis in Mice
Source: Front Nutr. 2022 Jun 9;9:918696. doi: 10.3389/fnut.2022.918696 (PMC9241564; doi:10.3389/fnut.2022.918696)
Supplement: Supplementary file 1 [file Data_Sheet_1.docx]

Supplementary Table 1. Basic nutritional composition of cooked adzuki bean (g/100g).

| Protein | 23.63±0.00 |
| --- | --- |
| Fat | 1.86±0.09 |
| Carbohydrate | 59.88±0.42 |
| Crude fiber | 4.63±0.04 |
| Sucrose | 0.310±0.003 |

Data are expressed as mean ± standard deviation (n = 3).

Supplementary Table 2. Composition of experimental diets

| Ingredient (g/kg)^a^ | LFD | HFD | HFD-CAB^c^ |
| --- | --- | --- | --- |
| Cooked adzuki bean | 0.00 | 0.00 | 150.00 |
| Casein, 80 Mesh | 189.56 | 258.45 | 220.3 |
| L-Cystine | 2.84 | 3.88 | 3.88 |
| Corn Starch | 479.79 | 0.00 | 0.00 |
| Maltodextrin 10 | 118.48 | 161.53 | 58.88 |
| Sucrose | 65.21 | 88.91 | 88.41 |
| Cellulose, BW200 | 47.39 | 64.61 | 59.94 |
| Soybean Oil | 23.70 | 32.31 | 28.42 |
| Lard | 18.96 | 316.60 | 316.60 |
| Mineral Mix | 9.48 | 12.92 | 12.92 |
| Dicalcium Phosphate | 12.32 | 16.80 | 16.80 |
| Calcium Carbonate | 5.21 | 7.11 | 7.11 |
| Potassium Citrate, 1 H_2_O | 15.64 | 21.32 | 21.32 |
| Vitamin Mix, V10001 | 9.48 | 12.92 | 12.92 |
| Choline Bitartrate | 1.90 | 2.58 | 2.58 |
| FD&C Yellow Dye #5 | 0.04 | 0.00 | 0.00 |
| FD&C Blue Dye #1 | 0.01 | 0.06 | 0.00 |
| Total | 1000 | 1000 | 1000 |
| % Energy and source^b^ | | | |
| Protein | 20 | 20 | 20 |
| Carbohydrate | 70 | 20 | 20 |
| Fat | 10 | 60 | 60 |
| Total | 100 | 100 | 100 |

^a^ When cooked adzuki bean is added to animal feed, the diet must be adjusted to maintain the original composition of cooked adzuki bean and ensure the consistency of energy intake. To equalize protein, carbohydrate, sucrose, fiber, and fat content (g/kg diet) between the HFD and HFD-CAB diets, casein, maltodextrin, sucrose, cellulose, and soybean oil are reduced in the HFD-CAB diet. Thus, the total macronutrient content between the HFD and HFD-CAB groups are equivalent.

^b^ The total energy and the fraction of energy obtained from fat, carbohydrate, and protein in the HFD and HFD-CAB groups are equal. Specifically, The HFD and HFD-CAB diets get 60% of calories from fat, 20% from carbohydrate, and 20% from protein. The energy density of the HFD and HFD-CAB diets are both 5.24 total kcal/g.

^c^ LFD, normal control diet; HFD, high-fat diet; HFD-CAB, high-fat diet supplemented with cooked adzuki bean.
